# Subchronic toxicity study of standardized methanolic extract of *Mitragyna speciosa* Korth in Sprague-Dawley Rats

**DOI:** 10.3389/fnins.2015.00189

**Published:** 2015-06-17

**Authors:** Mohd U. Ilmie, Hasnan Jaafar, Sharif M. Mansor, Jafri M. Abdullah

**Affiliations:** ^1^Department of Neurosciences, School of Medical Sciences, Universiti Sains MalaysiaKota Bharu, Malaysia; ^2^Department of Pathology, School of Medical Sciences, Universiti Sains MalaysiaKota Bharu, Malaysia; ^3^Centre for Drug Research, Universiti Sains MalaysiaGeorgetown, Malaysia; ^4^Centre for Neuroscience Services and Research, Universiti Sains Malaysia, Jalan Hospital Universiti Sains MalaysiaKota Bharu, Malaysia; ^5^Hospital Universiti Sains Malaysia, Jalan Hospital Universiti Sains MalaysiaKota Bharu, Malaysia

**Keywords:** standardized methanolic extract, *Mitragyna speciosa*, subchronic toxicity, oral administration, Sprague-Dawley rats

## Abstract

*Mitragyna speciosa* Korth, or better known as ketum, has long been used by traditional folk around Southeast Asia to prevent fatigue from working under hot tropical weather and as a replacement of opium, which can then cause addiction. To date, no findings have been reported of the toxic effect of ketum subchronically (28 days). Hence, the aim of this study was to investigate the toxicity of subchronic effect of standardized methanolic extract of ketum (SMEMS) in Sprague-Dawley rats. Rats were orally administered with 100, 200, and 500 mg/kg of SMEMS for 28 days. Body weights were recorded daily. They were terminated at day 28 to obtain data for hematology, biochemistry, and histopathology of the brain, liver, kidney, lung, heart, sciatic nerve, and spinal cord. The SMEMS affected body weight compared to control group. Biochemistry findings showed that liver and kidney were affected with the abnormal values in AST, creatinine, globulin, glucose, total protein, and urea. However, SMEMS produced toxic effect more to liver, kidney, and lung than other organs as observed histopathologically. The results suggested subchronic exposure of ketum is toxic to the physiology of the animals.

## Introduction

*Mitragyna speciosa* Korth, or known as ketum, in Malaysia belongs to Rubiaeceae (Idid et al., 1998), a coffee family. Ketum is an indigenous plant of Thailand and northern penisular Malaysia used traditionally in folk medicine, although it has been reported to cause addiction (Chittrakarn et al., 2008). Ketum has been traditionally used in Malaysia and Thailand as medicinal substance. Originally, local people used it to alleviate pain, coughing, or diarrhea. It is also used to prevent fatigue (Suwanlert, 1975). The leaf has been used in Thailand for its opium-like effect (Burkill et al., 1935). In addition to being used in its own right, it is often used to replace opium when opium is not available.

It was evidenced that mitragynine, the main alkaloid of ketum, at the cellukar level inhibits neurotransmitter release from the nerve endings of vas deferens, partly through the blockade of neuronal Ca^2+^ channels (Matsumoto et al., 2005). It is postulated that neuronal Ca^2+^ channel-blocking effect of mitragynine is the main mechanism for the analgesic and several other physiological actions of mitragynine. Other action, mitragynine was shown to inhibit forskolin-stimulated cAMP formation in NG108-15 cells *in vitro* (Tohda et al., 1997). Physiologically, ketum has shown to have antinociceptive, anti-inflammatory, gastrointestinal, and neurophysiological effects (Kumarnsit et al., 2006; Mossadeq et al., 2009; Idayu et al., 2011).

Acute administration of alkaloid extract of ketum significantly resulted in dose-dependent decreases in food and water intake in rats. Furthermore, prolonged suppressing effects were observed following administration of the ketum extract for 60 days and also significantly suppressed weight gaining (Kumarnsit et al., 2006). Standardised methanolic extract of ketum has been reported to increase blood pressure after an hour of administration. High dose of the extract also induced acute severe hepatotoxicity and mild nephrotoxicity. However, the extract has no effect on body weight, food and water consumption, absolute and relative organ weight, and hematology test (Harizal et al., 2010). The present study was designed to determine the toxic effects of standardized methanolic extract of *Mitragyna speciosa* (SMEMS) in subchronic exposure to the body to add to the information on period-influence exposure to the rodents.

## Materials and methods

### Plant material

Leaves of this species were collected from natural sources in Jengka, Pahang, Malaysia. The identification of this species was done by Forest Research Institute Malaysia (FRIM) and kept in Universiti Sains Malaysia (USM) herbarium with voucher number USM 11074. This plant was restricted to research purposes only.

### *Mitragyna Speciosa* korth methanolic extraction and standardization

The leaves of the plant were collected and thoroughly washed with distilled water to remove the dirt. The wet leaves were weighed and then dried in an oven at 50°C for 12 h. During this time, the leaves were periodically turned over to provide uniform drying. The dried leaves were ground to fine powder by a mill machine and the powder was weighed. Then, 100 g of the powder was exhaustively soxhlet extracted in methanol (100% v/v) by using an extractor and condenser (Ace Soxhlet Extractor 6730, Condenser 6740, Quick Fit, England) for 4 h at 60°C. Next, the extract was concentrated under reduced pressure at 40°C using a rotary evaporator. Then, it was further concentrated by allowing it to stand overnight in an oven at 30°C to remove any trace of methanol. The final product yielded 20 g of a dark green extract which was then screened for the presence of the alkaloid mitragynine using GC-MS. The extract produced was standardized with reference to the amount of mitragynine content using validated GC-MS method. Dried extract was stored at 4°C until further use (Amresh et al., 2007).

### Animals

Adult male Sprague-Dawley rats were weighing 50–60 g and aged 4 weeks were obtained from the breeding colony of the Animal Research and Service Centre (ARASC) USM. The animals were fed with standard commercial food pellets and water *ad libitum.* They were housed in a temperature-controlled room at 25 ± 2°C with a relative humidity of 50 ± 5% and a 12 h light/12 h dark cycle. All the experiments were performed with the approval of Animal Ethics Committee of USM.

### Drugs administration

SMEMS was suspended in distilled water homogenously to act as a vehicle. Six rats per group were administered with 100 (MS100), 200 (MS200), and 500 (MS500) mg/kg in each group while the control group received distilled water. Rats underwent administration for 28 days daily via oral route (OECD, 2008). Body weight was recorded daily starting from the 1 day administration until the end.

### Specimen collection

On the last day (day 28), the rats from each group were anesthetised by chloroform, and the blood was taken using cardiac puncture procedure. Some of the blood collected was put in a plastic test tube containing anticoagulant EDTA and some part used for serum collection (biochemical analyses). Then, the rats were decapitated (termination) by guillotine and dissected to obtain brain, lung, heart, kidney, liver, sciatic nerve, and spinal cord for histology purposes.

### Biochemical analysis

The blood samples that were collected during termination were centrifuged at 3000 rpm for 15 min to collect the serum. The serum samples then were aspirated off and frozen at −80°C. They were analyzed for the determination of albumin, alanine aminotransferase (ALT), aspartate aminotransferase (AST), cholesterol, creatinine, glucose, phosphate, globulin, urea, total protein, and lactate dehydrogenase (LDH). Biochemical analyses were carried out with an automatic chemistry analyser (Hitachi 902, Japan; Reagent by ROCHE Diagnostic GmbH, Germany).

### Haematology

The blood samples that were collected underwent blood count using the automatic hematology system (BC-3000 Plus, Mindray, Shenzhen, China) to evaluate red blood cell (RBC), white blood cell (WBC), hemoglobin (HGB), haematocrit (HCT), mean corpuscular volume (MCV), mean corpuscular hemoglobin (MCH), mean corpuscular hemoglobin concentration (MCHC), and platelet (PLT).

### Histopathology

#### Tissue preparation

The method of tissue preparation was adapted from Jain et al. (2008). The tissues obtained during the termination process were fixed in 10% formalin, dehydrated in gradual ethanol (50–100%). After that, the tissues were cleared using xylene and embedded in paraffin. The process continued with the sectioning process. All tissues were sectioned with 5 μm of thickness using microtome and transferred into a 40°C water bath for fishing process. The sections were fished by glass slides and then allowed to dry on the slide warmer.

#### Haematoxylin and eosin (H&E) staining

The H&E staining protocol followed a standard protocol of the Department of Pathology, USM. The staining process was started by soaking the sections in xylene twice for 2 min each. The process continued by soaking in absolute alcohol, 90% and 80% alcohol for 2 min each. Then, the slides were rinsed with tap water and were soaked in haematoxylin for 20 min. After that, the slides were rinsed again under running tap water. Then, they were dipped in acid alcohol for 3 s and were rinsed again for 5 min under tap water. Next, the slides were dipped in ammonia water for 10 s and rinsed for 2 min. The slides were then soaked in eosin for 2 min. The process continued by soaking the slides in 80, 90%, and absolute alcohol for 2 min each. The last step was soaking the slides in xylene twice for 2 min each. The sections were covered by cover slip and observed under microscope.

### Statistical analyses

Statistical analysis for body weight data was done by repeated measure ANOVA while Tukey's test was used for the *post-hoc* test. For hematology and biochemical analysis, data were analyzed using One-Way ANOVA. Differences between groups were considered significant if *p* < 0.05. All data points show the mean of standard error means (SEM).

## Results

### Body weight measurement

Subchronic exposure of SMEMS showed average body weight gains for MS100, MS200, and MS500 were 6.6, 7.9, and 5.2 g/day, respectively, compared to control of 7.8 g/day. MS500 showed the lowest weight gain compared to other dose regime groups and also the control group. There were significant differences between MS200 and other groups (*p* < 0.01, except for MS500 which *p* < 0.001).

### Biochemical analyses

Biochemistry data in Table 1 some of the parameters such as AST, creatinine, globulin, glucose, total protein, and urea showed significant differences when compared to respective group. Group MS100 differed significantly when compared to control group with *p* < 0.05 for AST. MS500 recorded significantly lower than MS100 for AST parameter. MS 200 and MS500 recorded significant differences in creatinine reading compared to the control group with *p* < 0.05 and *p* < 0.01, respectively. Among the groups, only MS200 recorded significantly high globulin readings compared to the control group with *p* < 0.05. In glucose, MS200 and MS500 were significantly higher than in the control group with *p* < 0.05. Total protein reading in MS200 was significantly higher than the control with *p* < 0.05. Lastly, both MS200 and MS500 showed significant differences in urea parameter when compared to both control and MS100 groups.

**Table 1:** **Biochemistry data of rats after subchronic administrations of SMEMS**.

**Parameter**	**Control (*n* = 6)**	**MS100 (*n* = 6)**	**MS200 (*n* = 6)**	**MS500 (*n* = 6)**
Albumin (g/l)	33.67 ± 0.422	34.50 ± 0.671	36.83 ± 1.302	35.50 ± 0.563
ALT (IU/L)	56.00 ± 1.862	85.33 ± 17.653	72.40 ± 7.325	61.00 ± 2.683
AST (IU/L)	156.33 ± 10.607	303.50 ± 55.438^#^	215.80 ± 19.368	100.50 ± 4.080^∅∅^
Cholesterol (mmol/l)	1.61 ± 0.045	1.59 ± 1.10 g	1.51 ± 0.132	1.46 ± 0.948
Creatinine (μmol/L)	49.50 ± 0.619	58.83 ± 1.108	63.17 ± 4.629^#^	65.00 ± 3.493^##^
Globulin (g/L)	30.50 ± 1.285	32.50 ± 1.177	40.17 ± 4.254^#^	35.50 ± 1.057
Glucose (mmol/l)	6.52 ± 0.294	7.47 ± 0.306	8.37 ± 0.390^#^	8.20 ± 0.218^#^
Phosphate (mmol/l)	2.56 ± 0.058	2.72 ± 0.092	2.87 ± 0.075	2.67 ± 0.081
LDH (mmol/l)	2471.67 ± 389.186	2189.33 ± 431.132	2587.20 ± 242.563	2650.83 ± 325.367
T. Protein (g/l)	64.17 ± 1.046	67.00 ± 1.366	77.00 ± 5.532^#^	69.60 ± 1.033
Urea (mmol/l)	6.967 ± 0.150	7.60 ± 0.348	9.23 ± 0.504^#^^∅^	9.30 ± 0.480^#^^∅^

Data indicate mean ± SEM.

ALT, alanine aminotransferase; AST, aspartate aminotransferase; LDH, lactate dehydrogenase.

# Statistically significant different compared to control, p < 0.05;

## Statistically significant different compared to control, p < 0.01;

∅ Statistically significant different compared to MS100, p < 0.05;

∅∅ Statistically significant different compared to MS100, p < 0.01.

### Haematology

Haematology results show that none of the groups differ significantly when compared to control for all parameters. Data presented were analyzed by using One-Way ANOVA test. The details are shown in Table 2.

**Table 2:** **Haematology data of rats after subchronic administration of SMMSE**.

**Parameter**	**Control (*n* = 6)**	**MS100 (*n* = 6)**	**MS200 (*n* = 6)**	**MS500 (*n* = 6)**
White Blood Cell (× 10^9^/L)	6.70 ± 0.071	7.23 ± 0.680	6.44 ± 0.745	6.03 ± 1.492
Haemoglobin (g/L)	14.34 ± 0.300	14.55 ± 0.393	15.38 ± 0.421	15.43 ± 0.259
Red Blood Cell (× 10^12^/L)	7.35 ± 0.140	7.36 ± 0.396	7.72 ± 0.336	7.62 ± 0.230
Haematocrit (L/L)	40.48 ± 0.876	40.86 ± 1.228	43.06 ± 1.421	43.05 ± 0.620
MCV (fL)	55.24 ± 1.490	55.93 ± 1.375	55.90 ± 0.719	56.78 ± 1.975
MCH (g/L)	19.48 ± 0.517	19.85 ± 0.552	19.92 ± 0.332	20.23 ± 0.453
MCHC (g/L)	35.38 ± 0.287	35.58 ± 0.125	35.70 ± 0.190	35.80 ± 0.549
Platelet (× 10^9^/L)	728.00 ± 70.379	907.00 ± 6.285	829.00 ± 46.242	850.50 ± 49.769

Data indicate mean ± SEM.

MCV, mean corpuscular volume; MCH, mean corpuscular hemoglobin; MCHC, mean corpuscular hemoglobin concentration.

### Histopathology

**Figure 1:**
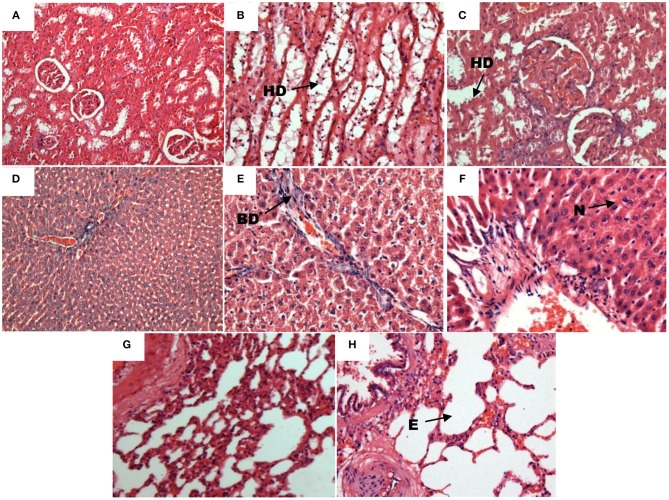
**Histopathology slides of affected organs after subchronic exposure to SMEMS. (A)** Kidney section from control (10X). **(B)** Hydropic degeneration present at the kidney's cortex of MS200 (20X). **(C)** Hydropic degeneration (**HD**) present at the kidney's medulla of MS500 (20X). **(D)** Liver section from control (10X). **(E)** Section of liver showing bile duct proliferation **BD** (20X). **(F)** Section of liver showing the presence of neurtrophil (**N**) from exposed group (20X). **(G)** Lung section from control (20X). **(H)** Section of lung showing emphysema (20X).

Histopathology results showed that brain, heart, spinal cord, and sciatic nerve were not affected by the subchronic exposure to SMEMS for all the doses when compared to normal. There were no abnormalities observed in the histopathology slides. However, the other organs such as liver, lung, and kidney were affected by SMEMS, as shown in Figure 1. Doses of 200 and 500 mg/kg affected kidney, liver, and lung significantly compared to the lowest dose and the vehicle group.

## Discussion

*M. speciosa* has long been used in treating illnesses traditionally. The present study has been conducted to gather information on the toxic effects of the subchronic consumption of the plant. Animals were exposed to SMEMS for 28 days via oral route. Throughout the administrations, daily body weight measurements were taken. On day 28, animals were euthanised to obtain blood and organs for biochemistry, hematology, and histopathology analyses. Dosages were justified based on previous study by Reanmongkol et al. (2007) that proved dose 100 mg/kg methanolic extract of ketum showed significant effect toward nociceptive response acutely. Thus, in this subchronic study we tested the same dose and increased to 200 and 500 mg/kg.

Animals in the group MS100 and MS500 showed significant body weight loss compared to the control group, while MS200 showed similarity in body weight gain compared to the control group. Kumarnsit et al. (2006) reported that ketum extract suppressed weight gain after 60 days of administration. Chittrakarn et al. (2008) documented ketum extract decreased the increment of body weight at doses of 200 and 400 mg/kg after 30 days of exposure. Mitragynine, the main biocompound of ketum, exerted an inhibitory effect on gastric acid secretion through opioid receptors. There is a probability that the mechanism of side effects such as anorexia and weight loss induced by mitragynine is related to the inhibitory effect on gastric acid secretion (Tsuchiya et al., 2002). Sabetghadam et al. (2013) reported a significant increase in the relative body weight of male rats treated with 28-day 1 mg/kg mitragynine. However, female rats treated with 28 days 100 mg/kg mitragynine recorded body weight decrease. Thus, high dose of ketum extract might affect the appetite at subchronic exposure.

This study also investigated biochemical analysis of the serum. Liver-related biochemicals such as AST, total bilirubin, and glucose in the present study showed statistically significant differences when compared to the control group. Our findings showed that the AST reading was significantly lower in rats that received the highest dose of ketum compared to rats that received the lowest dose of ketum. Significant differences were also observed between MS100 and control group. AST is one of the transaminases that are useful in evaluating muscle and liver damage in small and large animals. Its levels are expected to be less in the state of necrosis, as observed in the histopathology part of the present study. The findings showed that the level of creatinine in the groups MS200 and MS500 were higher significantly compared to that of the control group. Data from an acute toxicity study of ketum (dose of 1000 mg/kg) showed similar findings to the present study (Harizal et al., 2010). This biochemistry data was paralleled to our finding of kidney histopathology discussed below. The increase in serum creatinine demonstrates that the filtration process of the kidneys is deficient. Opioids such as morphine have been reported to cause renal damage by increasing the creatinine level (Atici et al., 2005). Another indicator of renal damage is the increase in the blood urea parameter. In the present study, the data show a high level of blood urea in MS200 and MS500 groups compared to that of control and MS100 groups. The findings also showed similarity to the acute toxicity study of ketum in Sprague-Dawley rats (Harizal et al., 2010) and rats treated with morphine chronically (Atici et al., 2005). Thus, *M. speciosa*, which possesses an opioid-like effect, is believed through chronic exposure to impair renal function or cause nephrotoxicity. Globulin was observed to be high in rats receiving 200 mg/kg ketum differed significantly from control. Globulins are important plasma proteins primarily associated with antibodies. Mitragynine showed significant increases in LDH, ALT, AST, and urea after being treated daily for 28 days with a subchronic dose of 100 mg/kg (Sabetghadam et al., 2013). This shows that the extract is toxic to the blood and might trigger the immune system to react toward that toxicity.

Subchronic administration of SMEMS not only caused damage to the liver as occurred in the acute toxicity studies of ketum from the histopathological data (Harizal et al., 2010; Kamal et al., 2012), but also caused damage to kidneys and lungs. At all doses, *M. speciosa* showed toxic effects for liver, kidneys and lungs. The observation by H&E staining showed the presence of blood vessels and neutrophils in MS500, portal inflammation, interphase necrosis, and bile duct proliferation in MS200 in the liver. Rats treated with chronic morphine suffered liver damage caused by focal vacuolar (fatty changes) degenerative change, with mononuclear cell infiltration in the hepatocytes (Sumathi and Niranjali Devaraj, 2009). Mild congestion of kidney blood vessels was also observed in MS100 and MS200. However, blood vessel congestion was nearly moderate in MS500. Hydropic degeneration was seen in the medulla and cortex regions of the kidney for MS100 and MS200, respectively. In MS500, hydropic degeneration was seen in both medulla and cortex regions. The effect of chronic morphine intoxication on rat kidneys showed mild tubular epithelial cell degeneration with cellular casts within the lumen of the tubules (Sumathi and Niranjali Devaraj, 2009). These effects of ketum extract on liver and kidney were similar to the effects of morphine (Atici et al., 2005). SMEMS exposed subchronically affected lungs by causing emphysema and the over-inflation of the alveoli. We believed that chemicals from the extract trapped in the alveoli later caused the inflammatory response, which will cause the alveolar septum to disintegrate known as septal rupture. Septal rupture leads to significant deformations of lung architecture (Nazari, 2002). Our unpublished data showed that SMEMS had fully blocked the long-term potentiation(LTP) in the same group of rat's hippocampal slices after subchronic exposure despite histopathology findings of the brain that did not show any apparent morphological changes. Li et al. (2012) reported that, long-term potentiation was impaired in xCT-deficient *sut* mice although no morphological changes were observed in the brain specimens stained with H&E and examined under the electron microscope. We speculate that the dosages applied in our experiments were insufficient to cause morphological alterations in the brain despite exhibiting changes in LTP.

In several parameters, the middle dose which is 200 mg/kg showed whether it was the highest or the lowest in giving such effects to certain results. This phenomenon can be explained as the hormetic response or U-shaped response. The hormetic response would reflect a decrease in the incidence of an adverse response such as disease/injury at low dose and an increase at higher dose (Calabrese and Baldwin, 2001).

## Conclusion

In conclusion, *M. speciosa* Korth is toxic to the rat upon subchronic exposure of 28 days. This toxicity can affect body weight, behavior, organs such as liver, kidney and lung and the blood. Generally, the higher doses in both MS200 and MS500 affected most of the parameters. Data from biochemical analyses and histopathology support each other and demonstrate that the plant is such an intoxicant material. The present study provides information primarily on whole-body toxicity effects at the subchronic level. Further study should be undertaken to seek the effect of chronic exposure to this plant in order to understand its potential for human use.

### Conflict of interest statement

The authors declare that the research was conducted in the absence of any commercial or financial relationships that could be construed as a potential conflict of interest.
